# Clinical and epidemiological profile of cases of deaths from stomach cancer in the National Cancer Institute, Brazil

**DOI:** 10.3332/ecancer.2014.445

**Published:** 2014-07-17

**Authors:** Maria Teresa dos Santos Guedes, José Paulo de Jesus, Odilon de Souza Filho, Raquel Malta Fontenele, Ana Inês Sousa

**Affiliations:** 1 Coordenadora de Enfermagem do Banco Nacional de Tumores e DNA/INCA, Mestre em Enfermagem/UNIRIO, Praça Cruz Vermelha, nº 23, Térreo, Centro-RJ, CEP 20230-130, Brazil; 2 Médico cirurgião e Chefe da Seção de Cirurgia Abdominopélvica do HC I/INCA, Brazil; 3 Médico cirurgião da Seção de Cirurgia Abdominopélvica do HC I/INCA, Doutorando da Universidade de São Paulo, Brazil; 4 Enfermeira, Mestre em Enfermagem pela Universidade Federal do Rio de Janeiro (UFRJ), Docente da Escola de Enfermagem Aurora de Afonso Costa da Universidade Federal Fluminense, Bolsista do Banco Nacional de Tumores e DNA/INCA, Brazil; 5 Enfermeira, Doutora em Ciências pela Fundação Oswaldo Cruz, Docente da Escola de Enfermagem Anna Nery da Universidade Federal do Rio de Janeiro (UFRJ), Brazil

**Keywords:** stomach neoplasms, adenocarcinoma, cross-sectional studies, medical oncology

## Abstract

**Introduction:**

Stomach cancer is the third most common cause of death worldwide, mainly affecting people with low socioeconomic status. In Brazil, we expect 20,390 new cases of stomach cancer in 2014, in both sexes, and according to the proportional distribution of the ten most prevalent types of cancer (except non-melanoma skin cancer) expected for 2014, this type of cancer was estimated to be the fourth most common in men and sixth in women.

**Aim:**

To investigate and analyse the clinical and epidemiological profile of deaths caused by stomach adenocarcinoma in patients enrolled in the National Cancer Institute, Brazil.

**Methods:**

Cross-sectional study, with samples which consisted of data from the medical records of deaths from stomach cancer, enrolled in the period from 1 February 2009 to 31 March 2012 and who had died as of 30 April 2012.

**Statistical Analysis Used:**

The Epi Info ®, version 7

**Results:**

We included 264 cases, mostly male. The mean age was 61.7 years. They were smokers, drinkers, white, and married, with elementary education and an income of one minimum salary. They had advanced stage disease (E IV), with symptoms characteristic of this phase, and the majority died within six months.

**Conclusion:**

The findings are similar to other studies. The advanced stage of the disease at the time of admission of the patients reflects the difficulty for users of the Unified Health System to access early diagnosis, demonstrating the need for efforts to identify groups and risk factors for the development of gastric cancer. Training of health professionals will facilitate planning and implementation of programmes for the prevention and control of disease, considering socioeconomic conditions, as seen in the sample, which is common among most users.

## Introduction

In Brazil, cancer is recognised as a public health problem and the country’s second leading cause of death from disease. In 2014, approximately 576,000 new cases of cancer are expected, including non-melanoma skin cancer, expanding the scope of the country’s cancer problem, especially considering that by 2050, the population of Brazil will reach 259.8 million [1].

This study focuses on stomach cancer (SC), the third most common cause of cancer deaths in the world for both sexes (723,000 deaths; 8.8% of the total), according to GLOBOCAN 2012. In Brazil, 20,390 new cases of SC are expected in 2014, in both sexes. Based on the proportional distribution of the ten most common types of cancer (excluding non-melanoma skin cancer) expected in 2014, SC is estimated to be the fourth most common type among men and the sixth among women [1–3].

SC presents a heterogeneous aetiology, including tobacco use, alcoholism, a diet high in sodium, nitrate, and nitrites that convert into nitrosamines with direct mutagenic properties, infection by the *Helicobacter pylori* bacterium involved in 60% of gastric adenocarcinomas, a diet low in fibre, vitamins and mineral salts from fresh fruits and vegetables, obesity, and a sedentary lifestyle. SC is rare in people under 45 years of age and more frequent in men. About two-thirds of all cases occur in less-developed countries, affecting primarily those with lower socioeconomic status [4, 5].

Given this, a study was conducted to determine and analyse the clinical-epidemiological profile of deaths caused by gastric adenocarcinoma in patients registered with the National Cancer Institute (INCA) between 1 February 2009 and 31 March 2012.

## Methods

A retrospective sectional study was conducted of a sample consisting of information from medical records of deaths attributable to gastric adenocarcinoma, recorded between 1 February 2009 and 31 March 2012, and which had been registered as deaths as of 30 April 2012. The research was conducted at Cancer Hospital I (HC I), in the INCA abdominal/pelvic surgery section. Cases of deaths of patients with a histopathological diagnosis different from adenocarcinoma or with records that could not be made available by the HC I registry archives were excluded from the study.

The registers used in the study were selected through the INCA internal virtual network by the International Classification of Diseases (ICD) code recorded in the virtual registry of patients. The deaths were identified through the absolute system.

The study variables were sex, age group, aggregate place of birth for regions of Brazil, level of education, marital status, monthly income, skin colour (based on the Brazilian Institute of Geography’s classification), history of alcoholism and tobacco use, symptoms reported by the patient, time of onset of symptoms, performance status (scale with criteria used by doctors and researchers to assess how neoplastic disease affects the daily living abilities of the patient, and to help in the assessment of how a patient’s disease is progressing, available at http://www.ecog.org/general/perf_stat.html) based on the Eastern Cooperative Oncology Group (ECOG) [6] stage assigned in the registration, blood type and RH factor, tumour location in the stomach, classification of the microscopic aspect based on Lauren [7], classification of the macroscopic aspect based on Borrmann [8], presence of signet ring and *Helicobacter pylori* cells, histological type and degree of cellular differentiation, INCA registration date, classification of malignant tumours (TNM) based on the seventh edition of the International Union Against Cancer-American Joint Committee on Cancer [9], types of oncological treatments administered, and death date. The statistical calculations were performed with the Epi Info public domain software, version 7, made available by the US Centers for Disease Control and Prevention.

The research project was submitted to the INCA research ethics committee for analysis and was approved by licence 189.849/12, authorising exemption from informed consent given that it involved collection and analysis of secondary data from the records of patients who died, without invasive procedures, administration of pharmaceuticals, or exposure of body images. Since individuals could not be identified, the confidentiality of the data collected was preserved as they were analysed anonymously and presented in aggregate form.

## Results

Between 1 February 2009 and 31 March 2012, 661 patients were registered with stomach tumours in the INCA-HC I abdominal/pelvic surgery section. From these, 300 cases of death were recorded (45.3%) up to 20 April 2012. Following application of the selection criteria, 13 of the 300 deaths were excluded because they did not involve gastric adenocarcinoma (TNE, GIST, non-Hodgkin lymphoma or squamous-cell carcinoma originating in the oesophagus with gastric proximal third invasion). Twenty-three cases were excluded for lack of access to records. This left a final sample of 264 patients.

Males predominated (64.4%), with age ranging from 24 to 90 years (mean = 61.7 years, median = 63, standard deviation = 14.5), while the majority (*n* = 117, 44.3%) were age 66 or older. 145 participants (54.9%) were reported to be white. The predominant place of birth was southeastern Brazil (72.4%). There were also six foreign patients (2.3%), from Argentina and Portugal. Most patients were married or reported cohabitation (*n* =173, 65.5%). The most common level of education was elementary education (*n* = 151; 57.2%), with family income of up to 01 minimum wage for 94 (49.2%).

In terms of clinical aspects, 164 patients (62.1%) were smokers. Most (*n* = 143; 53.3%) consumed alcoholic beverages. 119 (45.1%) of the patients presented with early symptoms of the disease between three and six months prior to the date of INCA registration. Performance Status 1 was predominant on the date of registration in 51.1% of patients (*n* = 135). It was noted that 90 records (34%) did not indicate blood type and RH factor, but of the 174 cases that did contain this information, blood type A positive (*n* = 83; 31.4%) was the most common (Table 1).

*Helicobacter pylori* infection was not mentioned in 163 cases (61.7%). Of the remaining 101 cases, 40 were positive for the bacteria. Poorly differentiated adenocarcinoma was the histological type for 178 patients (67.42%). Signet ring cells were present in 131 cases (49.62%). In the Lauren classification for microscopic aspects, the diffuse type was most common (*n* = 144, 54.5%). With regard to the macroscopic aspect, 140 patients (53.0%) presented with Borrmann type III. 159 (60.2%) were advanced, at stage IV. With respect to tumour location in the stomach, there was predominant involvement of the medial and distal third observed in 78 patients (29.5%), followed by the distal third in 62 cases (23.5%), and 45 cases (17%) presented with the disease in all three thirds (Table 1).

In terms of the time of onset of symptoms, 45.1% of patients (*n* = 119) reported the onset within a period ranging from three to six months prior to the date of admission to the institution. Among the symptoms reported by each patient at the time of INCA registration, there was a predominance of three or four symptoms (*n* = 133; 50.4%), the most common being weight loss for 45.52% (*n* = 117), epigastric pain for 40.0% (*n* = 103), and vomiting in 27.2% (*n* = 70) of cases.

As for the frequency and percentages of treatments administered, of the 264 cases analysed, 84 patients died before receiving treatment due to very advanced disease. Of the 180 cases treated, most (*n* = 69) underwent curative surgery. Of these 69 cases, 23 received neoadjuvant (*n* = 16) or adjuvant (n = 7) chemotherapy. Another 69 patients underwent palliative surgery (*n* = 18) or surgical staging (*n* = 51), combined with chemotherapy. Only one patient qualified for endoscopic resection, and underwent subsequent chemotherapy. The remaining 41 cases received palliative chemotherapy (*n* = 18), exclusive chemotherapy (*n* = 15) and chemotherapy combined with radiotherapy (*n* = 8).

In terms of time elapsed between INCA registration date and death (Table 2), it was noted that 103 patients (39.01%) died within six months of INCA registration. Another 100 died within 12 months, and only one patient survived 48 months.

## Discussion

The distribution of gastric cancer (GC) cases by age and gender followed the occurrence rate observed around the world and at the national level, which was almost twice as high in males (*n* = 170; 64.4%) than in females, and with occurrence peaks between 50 and 70 years, being an unusual condition at younger ages. However, a significant number of participants in this study were aged between 18 and 45 years (*n* = 33; 12.5%). The development of the disease at an early age may be due to germline mutation in the e-cadherin gene, autosomal dominant, which is responsible for hereditary gastric cancer syndrome (HGCS). A criterion to follow up on the suspicion of the syndrome is to diagnose GC before the age of 50 years [10–12].

Most participants were Caucasian (70.7%), and, in general, those with less education were mostly affected, in accordance with other articles published in Brazil, with the highest occurrences recorded for those who had either completed or failed to complete elementary school. The majority of patients in our study reported earning less than one minimum salary (*n* = 94; 49.2%). Consequently, those of lower socioeconomic status, measured by education and income, were the most affected, according to the results of other studies that strongly indicate twice the risk in the aforementioned social group. The association is mainly attributed to low socioeconomic status, which increases the likelihood of transmission and reinfection of *Helicobacter pylori* in household clusters with large families, poor sanitation, and less frequent use of antibiotics [13–16].

A lesser socioeconomic status may also be an indicator of poor access to health care and diets which are poor in fibre, vitamins, and mineral nutrients. Greater food availability leads to increased intake of fresh fruits, vegetables, and legumes, which could be a protective factor. The hypothesis that such a diet is healthier and thus protective is due to the fact that fresh fruits and vegetables possess vitamins with antioxidant properties, such as vitamins C, E, and beta-carotene [2, 4, 11].

Although one of the limitations of the study was the lack of information on the presence of *H. pylori* in the histopathology reports (163 cases without information; 61.7%), many studies explore the importance of the research of this bacterium, showing that most cases of GC are related to sporadic mutations in somatic cells, resulting from a long exposure of the gastric mucosa to inflammation caused by this bacterium. It is therefore essential to be alert to such a risk factor [4, 11].

GC’s history shows a decline, but it is still the world’s third leading cause of cancer deaths. Part of the explanation for this is due to a decrease in the predominance of infection by *H. pylori*, due to factors related to the use of refrigerators and improved food preservation techniques where, formerly, more salt, smoking, and other condiments were used [2, 5].

The study recorded the predominance of the medium and distal third in 53% of cases. This may be related to the fact that it is a preferred site of *H. pylori*, as described in the literature. However, other studies have shown a change in the presentation pattern of adenocarcinoma, of the distal to proximal regions, and with distinct histological features [13, 14], which was not found in our study.

For the duration of the symptoms, the beginning of the most intense symptoms ranging from three to nine months, and performance status 1 and 2 found in the majority (n = 195, 73.8%) at the time of admission of patient to treatment, it is evident the delay in seeking health-care services, or the difficulty to obtain a correct diagnosis and referral. It is therefore essential to improve the dissemination of health information at all health-care levels to professionals and the population alike.

Several studies have equally reported the interactions between personal lifestyle, such as smoking and drinking, and personal genetic factors and external agents, such as ionising radiation, chemical and biological carcinogens, as the causes for the development of most sporadic cancers, including GC [4, 5]. In this study, participants had, in most cases, been exposed to smoking and alcohol consumption risk factors.

Our study found 141 participants (53.3%) with blood group A+ or O+, which corroborates the findings that have already demonstrated this blood group’s relation with GC. Blood group A is associated with a high risk for SC when compared with other blood types. Blood group O is associated with a high risk for the development of peptic ulcer disease, which can lead to GC. A 50-year old database containing data on blood donors from Sweden and Denmark was analysed, and it revealed a 20% higher risk of SC in people with blood type A. A study in Korea investigating if there are specific gender differences in genotype associated to ABO for the risk of GC, found that genotypes AA and AO were significantly associated to GC exclusively in females, and exclusively for the diffuse type, suggesting that the association between blood group ABO and the risk of GC may vary according to gender and histological type [17–20].

ABO blood group antigens are the first human genetic markers and are explicit chemical components in the extracellular membrane of red blood cells and epithelial cells, including the gastrointestinal mucosa. The type of ABO group is defined by portions of carbohydrates (antigens A and B) of these membranes. Changes in the carbohydrate structures of the cell surface can change cell–cell and cell–extracellular matrix interactions, which can be important for tumour development. Also, a case-control study performed in Shanghai revealed that the proportion of infection by *H. pylori* in individuals of blood group A was significantly higher than in other blood groups. Furthermore, by adding the data from the results published by others, and associating the risk of GC with blood type, they found consistent evidence that the risk of GC in the type A group was higher than in the others [17, 19, 20].

Analysing the initial staging variables, the reported symptoms (weight loss and epigastric pain as the most common), the presence of signet ring cells, performance status, and survival after enrollment, we realise that the treatments offered have not been effective or even possible due to diagnosis obtained at advanced stages of the disease. The high predominance of the disease diagnosed at advanced stages III or IV (n = 216; 81.8%) is in the context of public institutions, which reflects the difficulty faced by patients who are dependent on the government’s Unified Health System (UHS) to obtain early diagnosis. And we know that the staging at diagnosis is the major prognostic determinant, despite other contributing factors, such as age over 50 years, gender, histological type, and low socioeconomic status [21–23].

We were able to observe all of these factors in the examined sample, where the majority (n = 231; 87.4%) were aged between 50 and 60 years, with performance status 1 or 2 (n = 195; 73.8%), histopathological diagnosis of slightly differentiated adenocarcinoma (n = 178; 67.42%), of diffuse type (n = 144; 54.5%), the presence of signet cells (n = 131; 49.62%), Bormann III or IV lesion (n = 232; 87.8%), and low education and low income in approximately over 75% of participants. Studies published since the 1990s had already shown that patients with Bormann type IV gastric cancer were often diagnosed at an advanced stage and with poor prognosis [24, 25].

Patients with Bormann type IV have poorer prognosis than patients with other tumour types because of the tardy diagnosis and subsequent detection of their advanced cancer stage. The more advanced tumour, the more frequent presence of lymph node metastasis, and the spread of malignant cells in the peritoneal cavity during surgery, cause reduced quality of resection [26, 27].

According to data from population-based registries compiled by the National Cancer Institute (“INCA”), the occurrence of GC has decreased, but mortality remains high. Also, there is a reference to the survival of 5 years in the west for approximately 30% of the cases in developed nations, and 20% of cases in developing nations. In the east, represented by Japan and South Korea, survival ranges from 50% to 70% due to screening and early detection programmes [13, 28, 29]. Our study has shown that 39.01% of participants (n = 103) died within 6 months after admission for treatment, 37.87% died after 12 months (n = 100), i.e. 5-year survival, similar to that of underdeveloped nations.

## Conclusion

This study corroborates the findings of other studies both nationally and internationally, differing only by a slight predominance in the number of cases of diffuse-type gastric adenocarcinoma. The advanced stage of the disease at which patients are admitted for treatment reflects the difficulty of users of the UHS to obtain early diagnosis at the different levels of health care.

This observation leads us to identify what efforts should be made, universally and equitably, to identify the groups and factors under risk of gastric cancer development, through the training of health professionals, thus enabling them to participate in the planning and implementation of gastric cancer prevention and control programmes. Policies addressing cancer should consider the socioeconomic conditions, which we have observed in our samples as common among the majority of users, in order to promote the dissemination of information about the disease, access to health care and known therapies.

This study is limited in terms of the collection and analysis of secondary data, mainly because of the inhomogeneity of entries in medical records, which limited the survey of other variables that would have otherwise enabled inferential statistical analysis and even descriptive analysis due to the number of records not reported, such as the cases of infection by *H. pylori*, per blood group or monthly income. Therefore, due care and the development of a broader prospective study are recommended.

## Competing Interests

The authors have no conflicts of interest to declare.

## Figures and Tables

**Table 1.** Distribution, based on clinical characteristics, of patients with gastric adenocarcinoma who died between February 2009 and March 2012 (*n* = 264).

Clinical Characteristics	n	%
**Tobacco use**		
Yes	**164**	**62,1**
No	95	36,0
Not reported	5	1.9
**Alcohol use**		
Yes	**143**	**54.2**
No	113	42.8
Not reported	8	3.0
**Performance status (ECOG)**		
0	36	13.6
1	**135**	**51.1**
2	60	22.7
3	16	6.1
4	2	0.8
Not reported	15	5.7
**Blood type and RH factor**		
O +	58	21.9
O –	5	1.8
A +	**83**	**31.4**
A –	2	0.7
AB+	3	1.1
AB –	22	8.3
B +	1	0.38
Not reported	90	34
***H. pylori***		
Yes	40	15.2
No	61	23.1
Not reported	**163**	**61.7**
**Histological type and degree of differentiation**		
Adenocarcinoma with little differentiation	**178**	**67.42**
Moderately differentiated adenocarcinoma	80	30.30
Highly differentiated adenocarcinoma	4	1.52
Not reported	2	0.76
**Presence of signet ring cells**		
Yes	**131**	**49.62**
No	127	48.11
Not reported	6	2.27
**Lauren classification**		
Diffuse	**144**	**54.5**
Intestinal	104	39.4
Mixed	4	1.5
Not reported	12	4.5
**Borrmann classification**		
I	4	1.5
II	14	5.3
III	**140**	**53.0**
IV	92	34.8
V	1	0.4
Not reported	13	4.9
**TNM stage**		
I	5	1.9
II	41	15.5
III	57	21.6
IV	**159**	**60.2**
Not reported	2	0.8
**Stomach area affected**		
Proximal third	30	11.4
Proximal + medial third	13	4.9
3 thirds	45	17.0
Medial third	34	12.9
Medial + distal third	**78**	**29.5**
Distal third	**62**	**23.5**
Proximal + distal third	1	0.4
Not reported	1	0.4
**TOTAL**	**264**	**100**

**Table 2.** Time elapsed between INCA registration date and death (*n* = 264).

Time (months)	Deaths within period	%
0–6	**103**	**39.01**
7–12	100	37.88
13–18	35	13.25
19–24	17	6.43
25–30	4	1.51
31–36	2	0.76
37–42	2	0.76
43–48	1	0.38
**Total**	**264**	**100**
